# A novel lncRNA-mRNA-miRNA signature predicts recurrence and disease-free survival in cervical cancer

**DOI:** 10.1590/1414-431X2021e11592

**Published:** 2021-09-20

**Authors:** Mengxiong Li, Xiaohui Tian, Hongling Guo, Xiaoyu Xu, Yun Liu, Xiulan Hao, Hui Fei

**Affiliations:** 1 Department of Obstetrics and Gynecology, The Seventh Affiliated Hospital of Sun Yat-sen University, Shenzhen, Guangdong, China

**Keywords:** Cervical cancer, Recurrence, Competing endogenous RNA, Molecular signature, FIGO staging

## Abstract

Cervical cancer (CC) patients have a poor prognosis due to the high recurrence rate. However, there are still no effective molecular signatures to predict the recurrence and survival rates for CC patients. Here, we aimed to identify a novel signature based on three types of RNAs [messenger RNA (mRNAs), microRNA (miRNAs), and long non-coding RNAs (lncRNAs)]. A total of 763 differentially expressed mRNAs (DEMs), 46 lncRNAs (DELs), and 22 miRNAs (DEMis) were identified between recurrent and non-recurrent CC patients using the datasets collected from the Gene Expression Omnibus (GSE44001; training) and The Cancer Genome Atlas (RNA- and miRNA-sequencing; testing) databases. A competing endogenous RNA network was constructed based on 23 DELs, 15 DEMis, and 426 DEMs, in which 15 DELs, 13 DEMis, and 390 DEMs were significantly associated with disease-free survival (DFS). A prognostic signature, containing two DELs (CD27-AS1, LINC00683), three DEMis (hsa-miR-146b, hsa-miR-1238, hsa-miR-4648), and seven DEMs (ARMC7, ATRX, FBLN5, GHR, MYLIP, OXCT1, RAB39A), was developed after LASSO analysis. The built risk score could effectively separate the recurrence rate and DFS of patients in the high- and low-risk groups. The accuracy of this risk score model for DFS prediction was better than that of the FIGO (International Federation of Gynecology and Obstetrics) staging (the area under receiver operating characteristic curve: training, 0.954 *vs* 0.501; testing, 0.882 *vs* 0.656; and C-index: training, 0.855 *vs* 0.539; testing, 0.711 *vs* 0.508). In conclusion, the high predictive accuracy of our signature for DFS indicated its potential clinical application value for CC patients.

## Introduction

Cervical cancer (CC) is one of the most common gynecological cancers worldwide. According to statistics using the Global Cancer Observatory database, there were approximately 570,000 cases of CC in 2018 (1). Among all countries, China contributed with the highest incidence burden (106,430 cases) (1). Although great advances have been made in the therapeutic options (such as surgery, radiotherapy, and chemotherapy), a considerable proportion of patients can develop relapse or metastasis, which may be the possible reason associated with a high mortality-to-incidence ratio in CC (about 30-50%) (1,2). Hence, how to early screen out patients at a high risk of poor prognosis may be a significant issue for gynecologists.

Accumulating evidence has demonstrated that the advanced International Federation of Gynecology and Obstetrics (FIGO) stage correlates with a high risk of recurrence and shorter length of 5-year survival (3). Thus, the FIGO staging system has been well recognized as a prognostic biomarker for CC in clinically. However, some studies indicate that the prognostic effectiveness of the FIGO staging system should be improved because survival differences could also be observed in patients within the same stage (4). Thus, identification of a more effective prognostic biomarker has become the main research focus. With the recent developments in sequencing technology and bioinformatics, identification of molecular biomarkers has gained much attention. Some molecular biomarkers were proven to have better predictive abilities than the FIGO staging system for survival in CC patients. For example, Zhao et al. (5) identified a prognostic signature that consisted of five protein-encoding messenger RNAs (mRNAs) (ITM2A, DSG2, SPP1, EFNA1, and MMP1) and found that only the molecular signature (P<0.05), but not the FIGO staging (P>0.05), was an independent indicator for the prediction of survival in CC patients. This conclusion was also seen in a study by Ju et al. (6) in which a five-mRNA signature (GALNTL6, ARSE, DPAGT1, GANAB, and FURIN) was shown to predict disease-free survival (DFS). Both univariate and multivariate Cox regression analyses revealed that a three-microRNA (miRNA) signature (miR-145, miR-200c, and miR-218-1) was significantly associated with overall survival (OS) of CC patients, while the FIGO staging was reported to be a significant prognostic factor only in univariate analysis (7). Mao et al. (8) screened nine long non-coding RNAs (lncRNAs) (ATXN8OS, C5orf60, DIO3OS, EMX2OS, INE1, KCNQ1DN, KCNQ1OT1, LOH12CR2, and RFPL1S) together as a single prognostic signature and demonstrated the predictive accuracy of this lncRNA signature for DFS was higher than that of the FIGO staging (area under the ROC (receiver operating characteristic) curve (AUC): training dataset, 0.793 *vs* 0.537; validation dataset, 0.780 *vs* 0.529; testing dataset, 0.742 *vs* 0.589). Based on the Harrell's Concordance Index (C-index), Cheng et al. (9) also showed the prediction ability of the six-lncRNA signature (LINC00619, FGF13-AS1, EMX2OS, WT1-AS, C9orf147, and LINC00908) for survival was higher than that of the FIGO staging (0.648 *vs* 0.516). However, the prediction power of the known signature remains relatively low (AUC<0.8 in most of studies) (6,), suggesting other candidate prognostic predictors are necessary for CC.

In recent studies on other cancers, we noticed that some scholars recommended integrating various RNA types to identify prognostic biomarkers. Also, they demonstrated that the multi-RNA-based classifier model was more effective to separate the survival of patients with different risks than lncRNA, miRNA, or mRNA alone (11,12), which has not been reported for CC patients. Therefore, the goal of this study was to develop a novel lncRNA-miRNA-mRNA prognostic signature to predict recurrence and DFS for CC patients.

## Material and Methods

### Data resource

One microarray dataset was downloaded from the Gene Expression Omnibus (GEO, http://www.ncbi.nlm.nih.gov/geo/) database under accession number GSE44001 (13) on April 8, 2019 (ID seen in Supplementary Table S1). GSE44001 dataset was run on the platform of Illumina HumanHT-12 WG-DASL V4.0 R2 expression beadchip (GPL14951, https://www.ncbi.nlm.nih.gov/geo/query/acc.cgi?acc=GPL14951). GSE44001 included 300 CC samples with recurrence (n=38, recurrent; n=262, non-recurrent) and DFS information and thus was set as the training dataset. During the corresponding period, matched RNA sequencing (RNA-seq; level 3, fragments per kilobase of exon per million fragments mapped value), miRNA-seq (level 3), and clinical data (recurrence status: n=28, recurrent; n=211, non-recurrent; and DFS time) of CC samples (ID seen in Supplementary Table S1) were also obtained from The Cancer Genome Atlas (TCGA, https://portal.gdc.cancer.gov/) database. TCGA data were obtained on the platform of Illumina HiSeq 2000 RNA Sequencing (https://tcga-xena-hub.s3.us-east-1.amazonaws.com/download/probeMap%2Fhugo_gencode_good_hg19_V24lift37_probemap) and selected as the testing dataset.

### Identification of differentially expressed RNAs

Gene symbol annotation was performed using HUGO Gene Nomenclature Committee (HGNC; http://www.genenames.org/) to identify gene classes (mRNAs, lncRNAs, and miRNAs). The RNAs with the median expression level of zero or only annotated in one dataset were deleted. The Linear Models for Microarray Data (LIMMA) package (v3.34.7; https://bioconductor.org/packages/release/bioc/html/limma.html) for R was used for differential analysis. |log_2_FC(fold change)|>0.5 and false discovery rate (FDR) <0.05 were defined as the threshold value to identify significantly differentially expressed mRNAs (DEMs), lncRNAs (DELs), and miRNAs (DEMis) between recurrent and non-recurrent samples of the GSE44001 dataset. The heatmap of DEMs, DELs, and DEMis was generated using the R packages of “pheatmap” (v1.0.8; https://cran.r-project.org/web/packages/pheatmap).

### Construction of a competing endogenous RNA (ceRNA) regulatory network

The ceRNA network was constructed based on the theory that lncRNAs can act as miRNA sponges and compete for miRNA binding to protein-coding mRNAs, influencing the negative regulation of miRNAs on the expression of mRNAs (14). Based on this hypothesis, the lncRNA-miRNA-mRNA ceRNA network was constructed following the steps below: 1) DIANA-LncBase (v2.0; http://carolina.imis.athenainnovation.gr/diana_tools/web/index.php?r=lncbasev2/index-predicted) was used to screen interactions between DELs and DEMis. Only the pairs with the opposite expression in DELs and DEMis were left; 2) starBase (v2.0; http://starbase.sysu.edu.cn/) database was used to retrieve the interactions between DEMis and DEMs. The starBase database included the prediction results from five databases (targetScan, picTar, RNA22, PITA, and miRanda). The interaction pairs predicted by any one database were included. Only the pairs with the opposite expression in DEMis and DEMs were retained; 3) lncRNA-miRNA-mRNA interaction axes were selected according to the intersected miRNAs interacted with DEMs and DELs; and 4) the ceRNA network was visualized using Cytoscape (v3.6.1; www.cytoscape.org/).

### Functional enrichment analysis

To understand the underlying functions of DEMs in the ceRNA network, Kyoto Encyclopedia of Genes and Genomes (KEGG) pathway and Gene Ontology (GO) biological process enrichment analyses were performed using the Database for Annotation, Visualization, and Integrated Discovery (DAVID) (v6.8; http://david.abcc.ncifcrf.gov). A P-value <0.05 served as the cut-off point.

### Establishment of a prognostic signature based on the ceRNA network genes

Univariate Cox regression analysis was performed to analyze the association between DEMs/DELs/DEMis in the ceRNA network and DFS using “survival” package in R (v2.41-1; http://bioconductor.org/packages/survivalr/). DEMs/DELs/DEMis that were significantly related with DFS (log-rank P-value <0.05) were entered into the multivariate Cox regression analysis to confirm their independence. The selected feature genes by the multivariate analysis (log-rank P-value <0.05) were used to fit a least absolute shrinkage and selection operator (LASSO)-Cox proportional hazards model (Cox-PH) model to further obtain an optimal gene panel using the Penalized package (v0.9-5; http://bioconductor.org/packages/penalized/). The risk score was built according to the expression levels of the RNAs (Exp_RNAs_) and their corresponding LASSO coefficients (Σβ_RNAs_): Prognostic risk score=Σβ_RNAs_ × Exp_RNAs._

Based on the median risk score, CC patients in the training dataset (GSE44001) were divided into two groups: the “low-risk” group and the “high-risk” group. Kaplan-Meier (K-M) survival curves were conducted using the “survival” package in R to identify the DFS differences between two groups. The ROC curve within 5 years was plotted and the AUC was computed to estimate the predictive ability of the risk score. To demonstrate the predictive accuracy of the risk score better than the FIGO staging for DFS prediction, several analyses were performed, including stratification with K-M curves, estimation of AUC for time-dependent ROC curves, and calculation of C-index for various survival models using survcomp package in R (http://www.bioconductor.org/packages/release/bioc/html/survcomp.html). The robustness of the prognostic signature for predicting DFS in CC patients was subsequently assessed in the testing dataset (TCGA).

## Results

### Identification of DEMs, DELs, and DEMis

**Figure 1:**
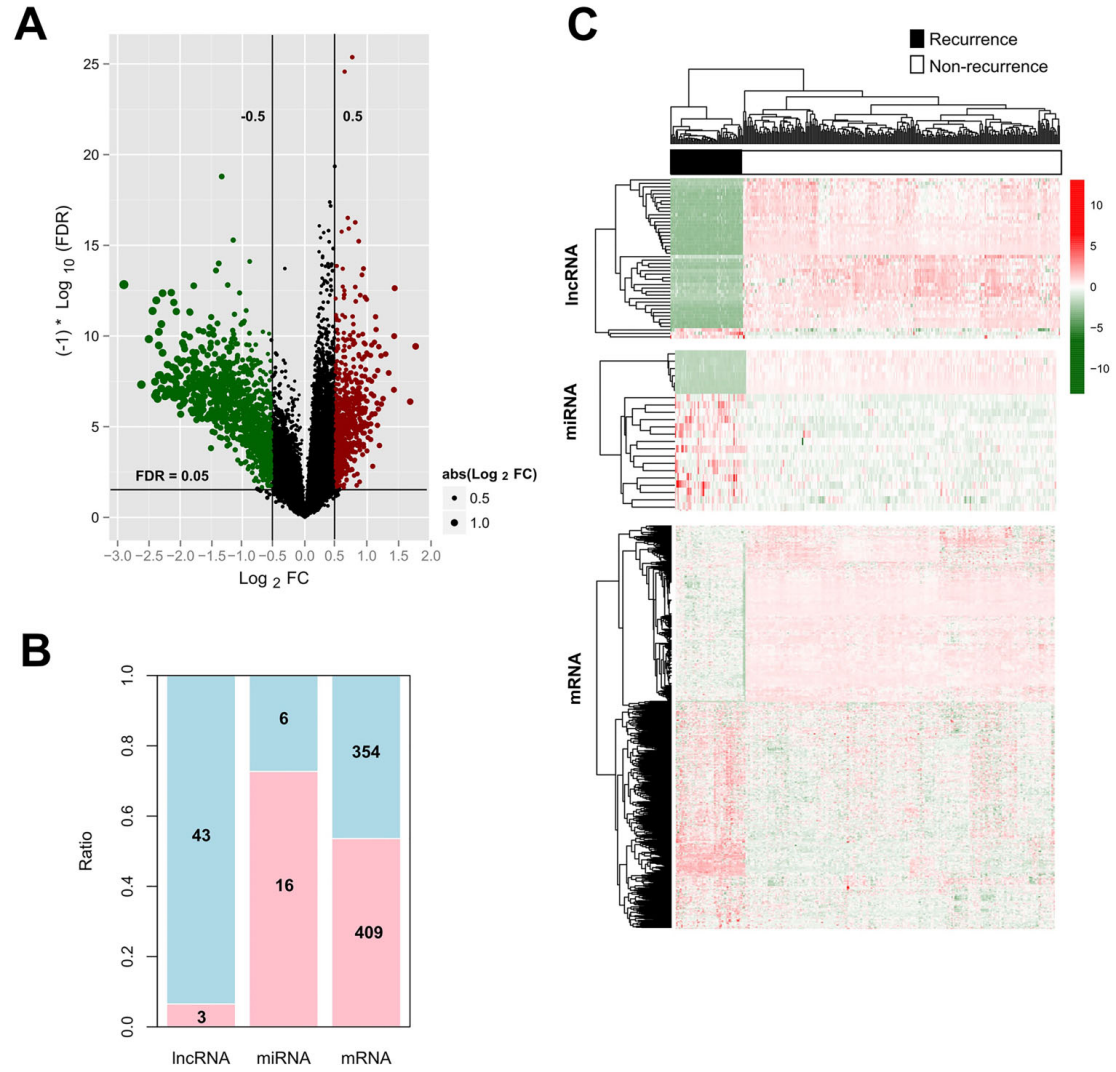
Identification of differentially expressed RNAs between recurrent and non-recurrent cervical cancer samples. **A**, Volcano plot to show the distribution of differentially expressed RNAs (green dots, downregulated; red dots, upregulated). FC: fold change; FDR: false discovery rate. **B**, Upregulated and downregulated number of differentially expressed mRNAs, lncRNAs, and miRNAs. **C**, Heat map of differentially expressed mRNAs, lncRNAs, and miRNAs. Red: high expression; green: low expression.

By HGNC annotation, 11,187 mRNAs, 249 lncRNAs, and 229 miRNAs were found in the GSE44001 dataset. According to the cut-off threshold (FDR<0.05 and |log_2_FC|>0.5), 763 DEMs (409 upregulated and 354 downregulated), 46 DELs (3 upregulated and 43 downregulated), and 22 DEMis (16 upregulated and 6 downregulated) were identified by comparing recurrent and non-recurrent CC samples (Figure 1A and B; Supplementary Table S2). The heat map showed that these DEMs, DELs, and DEMis can provide a clear separation for the recurrent and non-recurrent samples (Figure 1C).

### Construction of a ceRNA network

**Figure 2:**
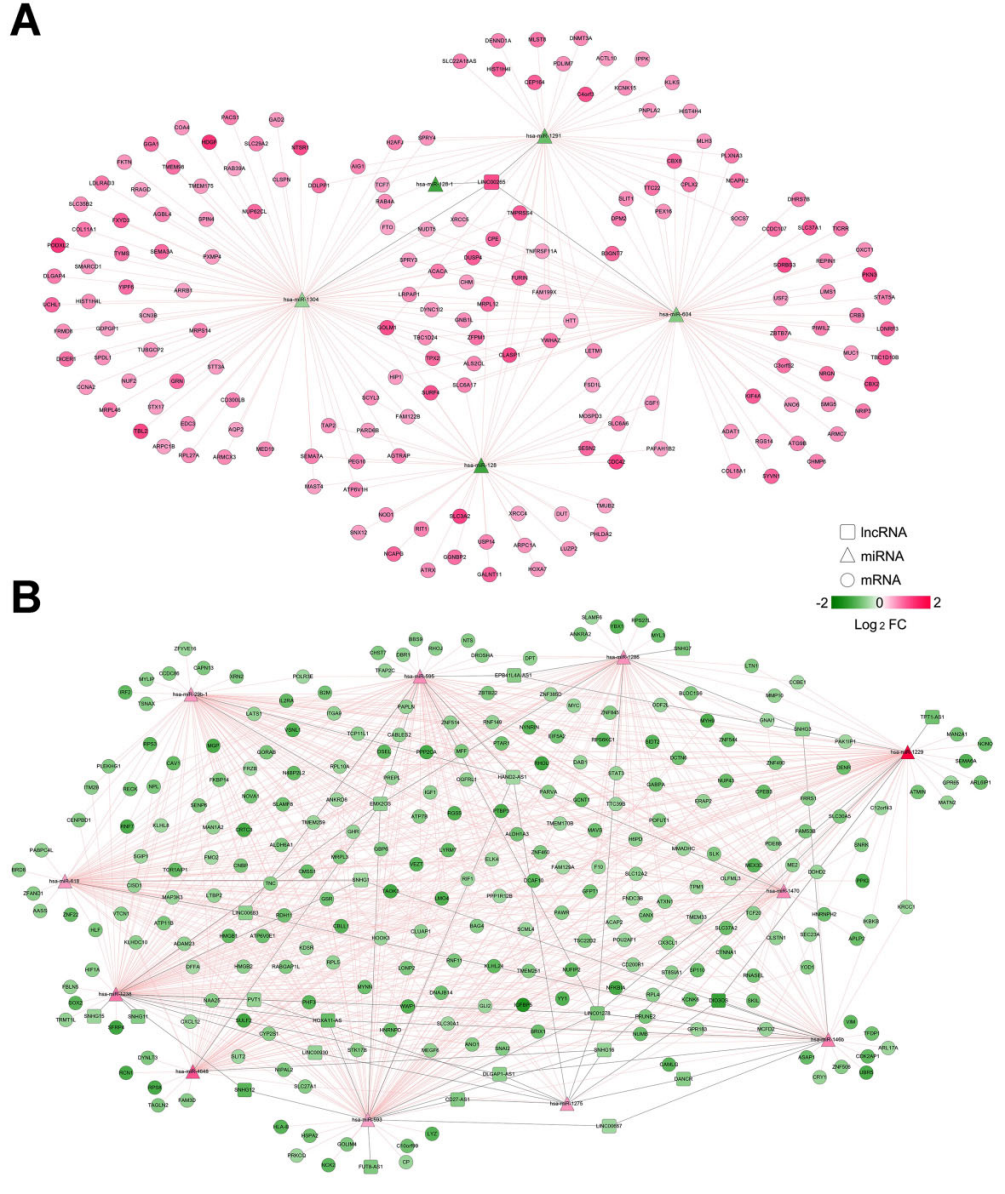
Construction of a competing endogenous RNA network among differentially expressed lncRNAs, miRNAs, and genes. **A**, Relationship pairs among upregulated lncRNA, downregulated miRNAs, and upregulated mRNAs; **B**, Relationship pairs among downregulated lncRNAs, upregulated miRNAs, and downregulated mRNAs. Red: upregulated; green: downregulated.

A total of 63 interaction pairs between 23 DELs and 15 DEMis were predicted by the DIANA-LncBase database (such as CD27-AS1-hsa-miR-1275, LINC00683-hsa-miR-146b, EMX2OS-hsa-miR-1238/4648, LINC00265-has-miR-128-1/604/1304, EMX2OS-hsa-miR-29b-1), while 1,038 paired interactions between 15 DEMis and 426 DEMs were predicted by the starBase database [such as hsa-miR-1275-ANKRD6 (ankyrin repeat domain 6), hsa-miR-595/4648-GHR (growth hormone receptor), hsa-miR-1238-FBLN5 (fibulin 5), hsa-miR-146b-ACAP2 (ArfGAP with coiled-coil, ankyrin repeat and PH domains 2), has-miR-128-1-ATRX (ATRX chromatin remodeler), hsa-miR-29b-1-MYLIP (myosin regulatory light chain interacting protein), hsa-miR-604-ARMC7 (armadillo repeat containing 7)/OXCT1 (3-oxoacid CoA-transferase 1), and hsa-miR-1304-RAB39A (RAB39A, member RAS oncogene family)]. According to the intersected miRNAs, an lncRNA-miRNA-mRNA ceRNA network was constructed (Figure 2).

### Functional enrichment analysis

**Figure 3:**
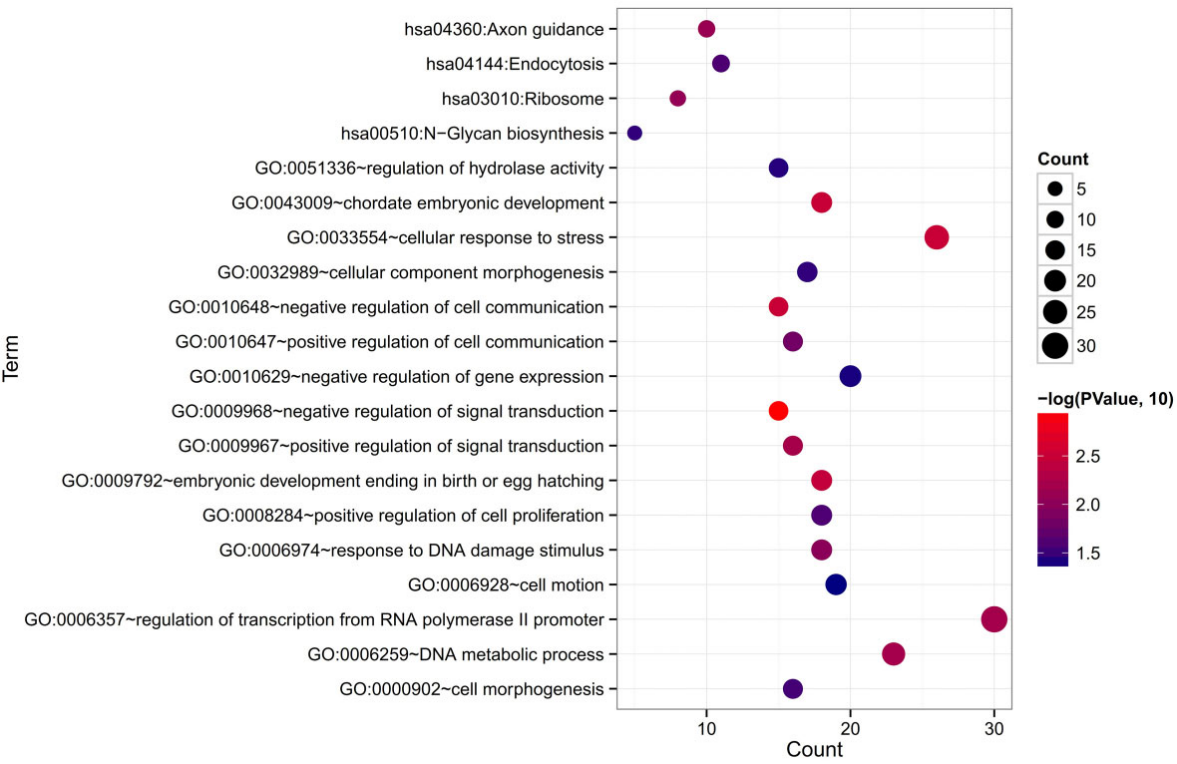
Functional enrichment results for genes in the competing endogenous RNA network. GO: Gene Ontology.

To further understand the functions of DEMs in the ceRNA network, functional enrichment analysis was performed. The results showed that 16 GO biological process terms were enriched, including GO:0033554∼cellular response to stress (ATRX), GO:0009967∼positive regulation of signal transduction (GHR), GO:0006259∼DNA metabolic process (ATRX), GO:0006974∼response to DNA damage stimulus (ATRX), GO:0010647∼positive regulation of cell communication (GHR), GO:0051336∼regulation of hydrolase activity (ACAP2), and GO:0006928∼cell motion (MYLIP) (Figure 3; Table 1). Also, four KEGG pathways were enriched, such as hsa04144:Endocytosis (ACAP2) (Figure 3; Table 1).

**Table 1:** Functional enrichment for genes in the ceRNA network.

Category	Term	P-value	Genes
GO BP	GO:0009968∼negative regulation of signal transduction	1.070E-03	CAV1, PAK1IP1, TAOK3, SOX2, NFKBIA, SOCS7, PAWR, FRZB, RGS14, ATXN1, PEG10, PPP2CA, RGS5, SKIL, IGFBP5
	GO:0033554∼cellular response to stress	3.092E-03	XRCC5, XRCC4, HMGB1, CLSPN, CAV1, HMGB2, ST8SIA1, RPS27L, CEP164, MLH3, TPM1, RPS3, AQP2, NONO, TYMS, SLK, FAM129A, CRY1, CCNA2, ATG9B, TAOK3, ATMIN, ATRX, HIF1A, RIF1, UBR5
	GO:0010648∼negative regulation of cell communication	3.131E-03	CAV1, PAK1IP1, TAOK3, SOX2, NFKBIA, SOCS7, PAWR, FRZB, RGS14, ATXN1, PEG10, PPP2CA, RGS5, SKIL, IGFBP5
	GO:0043009∼chordate embryonic development	3.217E-03	XRCC4, LIMS1, SYVN1, HTT, LMO4, GABPA, MYH9, GLI2, TPM1, YBX1, MAN2A1, HIF1A, SLC30A1, GFPT1, GRN, HOXA7, POFUT1, COL11A1
	GO:0009792∼embryonic development ending in birth or egg hatching	3.511E-03	XRCC4, LIMS1, SYVN1, HTT, LMO4, GABPA, MYH9, GLI2, TPM1, YBX1, MAN2A1, HIF1A, SLC30A1, GFPT1, GRN, HOXA7, POFUT1, COL11A1
	GO:0009967∼positive regulation of signal transduction	5.978E-03	MAVS, CAV1, F10, CSF1, KLK5, TAOK3, SOX2, IGF1, FURIN, SORBS3, HIF1A, NOD1, MAP3K3, SLC35B2, MLST8, GHR
	GO:0006357∼regulation of transcription from RNA polymerase II promoter	6.290E-03	HMGB1, HMGB2, CNBP, STAT5A, SOX2, NFKBIA, CBX2, PAWR, GLI2, SORBS3, HOXA7, SMARCD1, SKIL, MYC, TFDP1, BRD8, DNMT3A, ZBTB7A, TCF7, YY1, GABPA, IGF1, SNAI2, USF2, STAT3, ATXN1, MED19, HIF1A, IRF2, TFAP2C
	GO:0006259∼DNA metabolic process	6.387E-03	XRCC5, XRCC4, DNMT3A, CLSPN, HMGB1, HMGB2, DFFA, IGF1, RPS27L, CEP164, MLH3, NONO, ATRX, TYMS, SLK, CDK2AP1, PIWIL2, CRY1, MYC, XRN2, REPIN1, NYNRIN, DUT
	GO:0006974∼response to DNA damage stimulus	1.019E-02	XRCC5, XRCC4, HMGB1, CLSPN, HMGB2, RPS27L, CEP164, MLH3, ATMIN, RPS3, NONO, ATRX, TYMS, RIF1, SLK, UBR5, CRY1, CCNA2
	GO:0010647∼positive regulation of cell communication	1.534E-02	MAVS, CAV1, F10, CSF1, KLK5, TAOK3, SOX2, IGF1, FURIN, SORBS3, HIF1A, NOD1, MAP3K3, SLC35B2, MLST8, GHR
	GO:0008284∼positive regulation of cell proliferation	2.540E-02	COL18A1, XRCC4, CNBP, IL2RA, STAT5A, CSF1, SOX2, ST8SIA1, IGF1, GLI2, PRKCQ, NCK2, TNFRSF11A, HIF1A, GRN, EIF5A2, MYC, CCNA2
	GO:0000902∼cell morphogenesis	2.860E-02	COL18A1, PARD6B, PLXNA3, LIMS1, UCHL1, MYH9, GLI2, CXCL12, SLIT1, SLIT2, CDC42, SEMA6A, HIF1A, NUMB, CLASP1, SEMA3A
	GO:0032989∼cellular component morphogenesis	3.458E-02	COL18A1, PARD6B, PLXNA3, LIMS1, UCHL1, MYH9, GLI2, CXCL12, SLIT1, TPM1, SLIT2, CDC42, SEMA6A, HIF1A, NUMB, CLASP1, SEMA3A
	GO:0051336∼regulation of hydrolase activity	3.730E-02	CAV1, TBC1D10B, MYL3, ASAP1, RABGAP1L, FURIN, TPM1, RPS3, TBC1D24, NOD1, ACAP2, CHM, MLST8, MYC, HIP1
	GO:0010629∼negative regulation of gene expression	4.026E-02	DNMT3A, HMGB1, ZBTB7A, HMGB2, DICER1, SOX2, GABPA, CBX2, PAWR, GLI2, SNAI2, STAT3, ATXN1, SORBS3, WWP1, HOXA7, PIWIL2, IRF2, SKIL, MYC
	GO:0006928∼cell motion	4.298E-02	PLXNA3, VIM, IGF1, SCYL3, CX3CL1, MYLIP, MYH9, GLI2, CXCL12, SLIT1, TPM1, SLIT2, STAT3, SEMA6A, ARPC1B, NCK2, DAB1, HIF1A, SEMA3A
KEGG pathway	hsa04360:Axon guidance	7.635E-03	CDC42, NCK2, SEMA6A, PLXNA3, GNAI1, SEMA7A, SEMA3A, SLIT1, CXCL12, SLIT2
	hsa03010:Ribosome	8.594E-03	HNRNPH2, RPL27A, RPS27L, RPL5, RPL4, RPL10A, RPS8, RPS3
	hsa04144:Endocytosis	2.593E-02	CDC42, PARD6B, IL2RA, HSPA2, ARRB1, CHMP6, RAB4A, WWP1, ACAP2, ASAP1, HLA-B
	hsa00510:N-Glycan biosynthesis	3.436E-02	MAN2A1, STT3A, MAN1A2, DPM2, DOLPP1

GO BP: Gene Ontology (GO) biological processes; KEGG: Kyoto Encyclopedia of Genes and Genomes.

### Construction of a prognostic signature

Univariate Cox regression analysis detected that among 464 RNAs in the ceRNA network, 418 were significantly associated with DFS, including 390 DEMs (i.e., ANKRD6, WWP1, ACAP2), 15 DELs (i.e., EMX2OS, LINC00265), and 13 DEMis (i.e., hsa-miR-1275, has-miR-128-1, hsa-miR-29b-1, hsa-miR-604) (Supplementary Table S3). Of them, 29 (22 DEMs, two DELs, and five DEMis) were screened as the independent predictors of DFS by the multivariate Cox regression analysis. LASSO Cox regression model analysis further showed that 12 of these 29 RNAs were the optimal prognostic panel (2 DELs: CD27-AS1, LINC00683; 3 DEMis: hsa-miR-146b, hsa-miR-1238, hsa-miR-4648; 7 DEMs: ARMC7, ATRX, FBLN5, GHR, MYLIP, OXCT1, RAB39A) (Table 2). Hence, the risk score was built based on the expression of these 12 signature genes and their LASSO coefficients: risk score=(-0.2223297) × Exp_CD27-AS1_ + (0.403561456) × Exp_LINC00683_ + (1.275309117) × Exp_hsa-miR-146b_ + (-2.408082103) × Exp_hsa-miR-1238_ + (-0.574392794) × Exp_hsa-miR-4648_ + (0.848557903) × Exp_ARMC7_ + (0.868981201) ×Exp_ATRX_ + (-0.573439111) × Exp_FBLN5_ + (-0.457116812) × Exp_GHR_ + (-0.56842456) × Exp_MYLIP_ + (0.872944103) × Exp_OXCT1_ + (0.777716643) × Exp_RAB39A_.

**Table 2:** The 12-gene-based signature.

Type/Symbol	Expression	Multivariate Cox regression			LASSO coefficient
		HR	95%CI	P-value	
lncRNA					
CD27-AS1	Downregulated	0.799	0.541-0.981	2.610E-02	-0.2223297
LINC00683	Downregulated	1.856	1.125-3.062	1.554E-02	0.403561456
miRNA					
hsa-miR-146b	Upregulated	4.439	1.959-10.061	3.570E-04	1.275309117
hsa-miR-1238	Upregulated	0.339	0.105-0.969	4.103E-02	-2.408082103
hsa-miR-4648	Upregulated	0.888	0.431-0.931	4.733E-02	-0.574392794
mRNA					
ARMC7	Upregulated	3.074	1.639-5.766	4.650E-04	0.848557903
ATRX	Upregulated	3.285	1.518-7.108	2.533E-03	0.868981201
FBLN5	Downregulated	0.728	0.475-0.964	1.458E-02	-0.573439111
GHR	Downregulated	0.478	0.338-0.675	2.810E-05	-0.457116812
MYLIP	Downregulated	0.644	0.439-0.946	2.474E-02	-0.56842456
OXCT1	Upregulated	3.228	1.444-7.214	4.295E-03	0.872944103
RAB39A	Upregulated	2.495	1.294-4.810	6.333E-03	0.777716643

HR: hazard ratio; CI: confidence interval; LASSO: least absolute shrinkage and selection operator.

**Figure 4:**
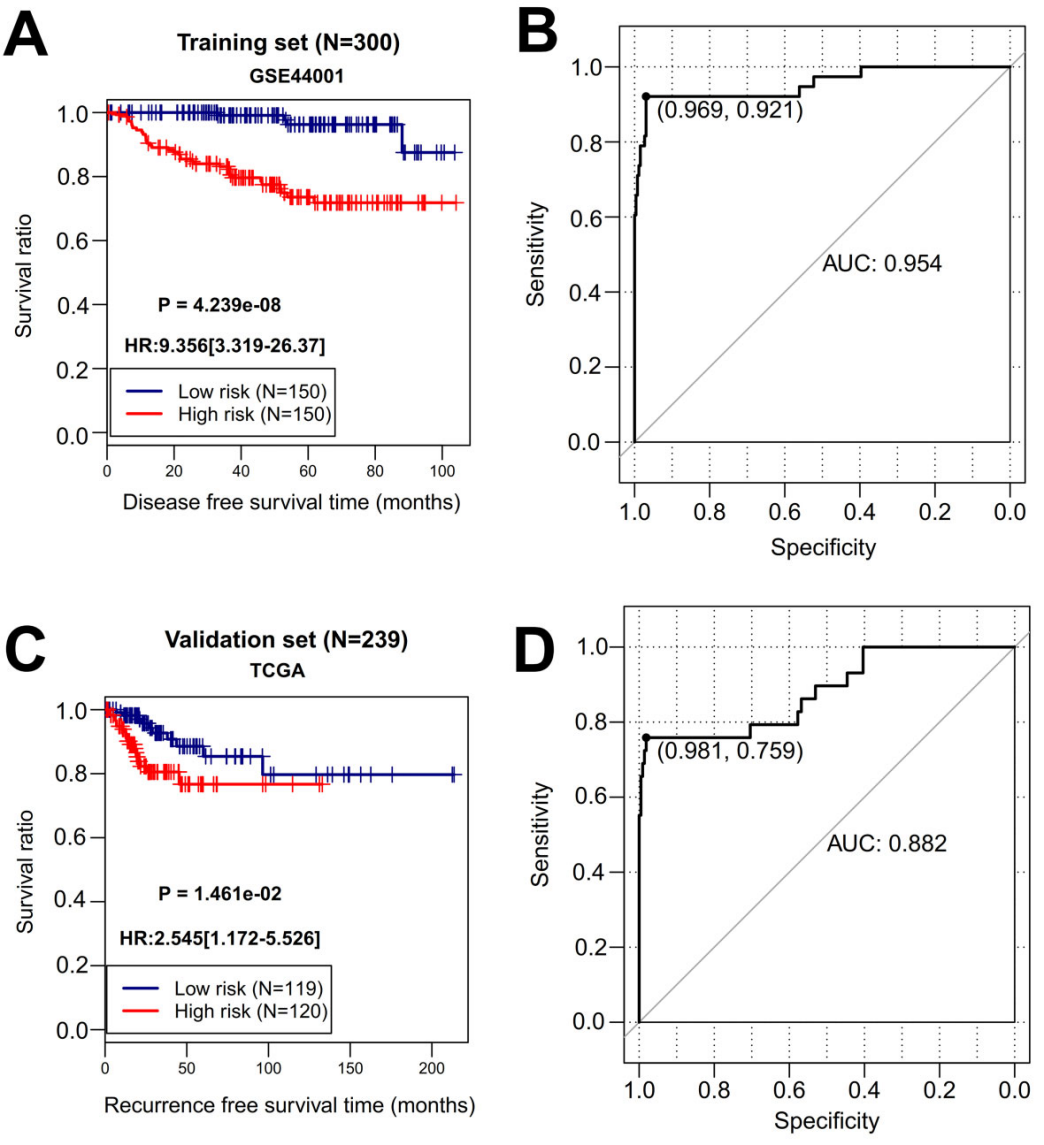
Prognostic evaluation of the 12-multi-RNA-based risk score for cervical cancer patients. **A**, Kaplan-Meier curve of the training dataset (GSE44001). **B**, Receiver operator characteristic curve of the training dataset (GSE44001). **C**, Kaplan-Meier curve of the testing dataset (TCGA). **D**, Receiver operator characteristic curve of the testing dataset (TCGA). AUC: area under the receiver operator characteristic curve; HR: hazard ratio.

**Figure 5:**
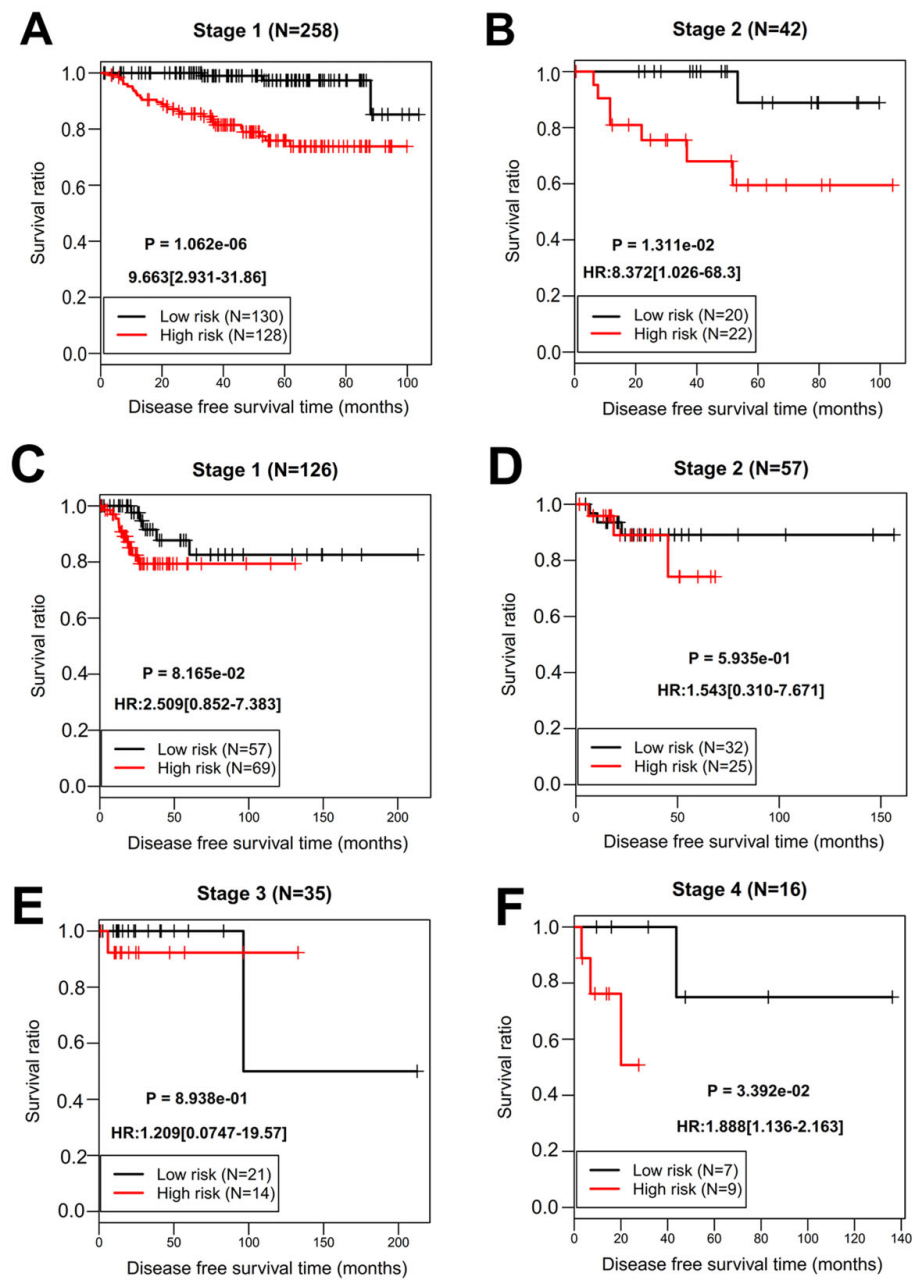
Stratification analysis according to the FIGO staging. Kaplan-Meier survival curve for **A**, patients with stage 1 (GSE44001); **B**, patients with stage 2 (GSE44001); **C**, patients with stage 1 (TCGA); **D**, patients with stage 2 (TCGA); **E**, patients with stage 3 (TCGA); **F**, patients with stage 4 (TCGA). HR: hazard ratio.

**Figure 6:**
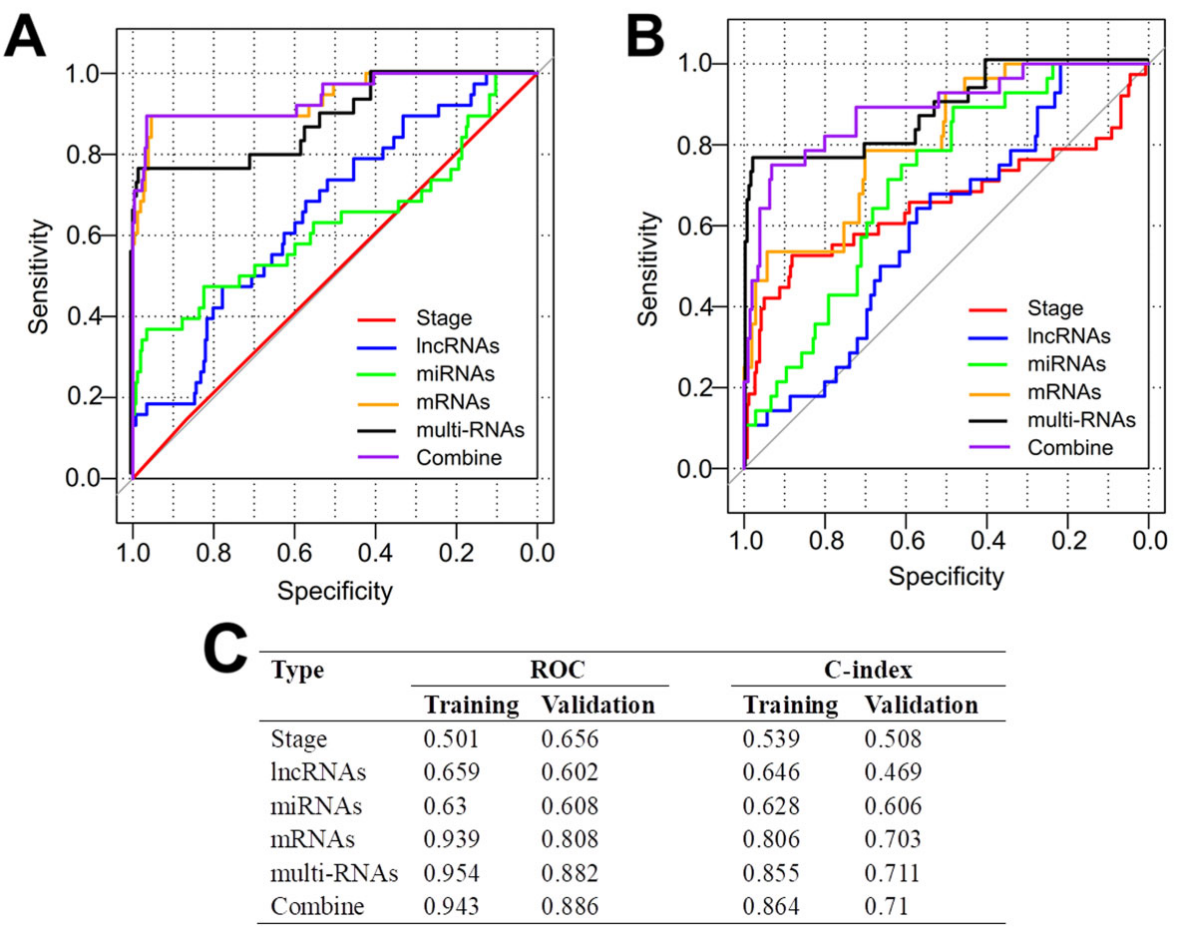
Comparison of the prognostic power among the risk score model, the FIGO staging, and mRNA, miRNA, lncRNA alone. **A**, Time-dependent receiver operator characteristic curves (ROC) using the training dataset (GSE44001). **B**, Time-dependent ROC curves using the testing dataset (TCGA). **C**, Calculated results for ROC and C-index.

The risk score was calculated for each sample in the training set. According to the median risk score, patients were divided into high- (n=150) and low-risk (n=150) groups (Supplementary Table S1). The recurrence rate of the patients in the high-risk group was significantly higher than that in the low-risk group (34/150, 22.7% *vs* 4/150, 2.7%; chi-squared=27.029, P<0.001). K-M survival curve analysis showed that the DFS time was significantly shorter in the high-risk group relative to the low-risk group [hazard ratio (HR)=9.356; 95%CI: 3.319-26.37; P=4.239e-08] (Figure 4A). ROC survival curve analysis revealed that this 12-gene signature exhibited a high accuracy to predict the 5-year DFS of CC patients, with the AUC of 0.954 (Figure 4B). Stratification analysis demonstrated that the DFS of patients with the same stage (1 or 2) could be significantly distinguished when they were divided into high- and low-risk groups by the risk score (Figure 5A and B). Time-dependent ROC curve (AUC=0.954 *vs* 0.501) and C-index (0.855 *vs* 0.539) analyses further confirmed that the predictive accuracy of the risk score was better than the FIGO staging for DFS prediction (Figure 6A and C). Furthermore, we also evaluated the prognostic robustness of this 12-gene signature by using the TCGA as the testing dataset. In line with the results of the training dataset, K-M survival curve analysis showed the high-risk group was correlated with poorer DFS compared to that of the low-risk group (HR=2.545; 95%CI: 1.172-5.526; P=1.461e-02) (Figure 4C). The AUC was 0.882 in the ROC survival curve analysis (Figure 4D). Stratification analysis showed a statistical difference in DFS between patients at high and low risk, although they all belonged to stage 4 (Figure 5F). The DFS in patients with stage 1 to 3 were not significantly different (Figure 5C-E). Time-dependent ROC curve (AUC=0.882 *vs* 0.656) and C-index (0.711 *vs* 0.508) analyses further confirmed the higher predictive accuracy of risk score for DFS compared with the FIGO staging (Figure 6B and C).

## Discussion

Although studies have investigated a molecular signature to predict the recurrence and DFS (or recurrence free survival, RFS) for CC patients (6,8), the known signature was mainly composed of 5 mRNAs (6), 9 lncRNAs (8), or 9 miRNAs (15). No studies focused on identification of a combined-RNA signature. In the present study, we attempted to develop a novel signature to distinguish the CC patients having a high risk to recurrence and poor DFS from those with a low risk based on the lncRNAs, miRNAs, and mRNAs in the ceRNA network. This analysis flow may be beneficial to identify some mechanism-clear, prognostic associative biomarkers. As expected, our identified prognostic signature included two lncRNAs (CD27-AS1, LINC00683), three miRNAs (hsa-miR-146b, hsa-miR-1238, hsa-miR-4648), and seven mRNAs (ARMC7, ATRX, FBLN5, GHR, MYLIP, OXCT1, RAB39A). Almost all of their related ceRNA interactive axis genes (CD27-AS1-hsa-miR-1275-ANKRD6, LINC00683-hsa-miR-146b-ACAP2, EMX2OS-hsa-miR-1238-FBLN5, EMX2OS-hsa-miR-4648-GHR, EMX2OS-hsa-miR-29b-1-MYLIP, LINC00265-has-miR-128-1-ATRX, LINC00265-hsa-miR-604-ARMC7/OXCT1, LINC00265-hsa-miR-1304-RAB39A) were associated with DFS except hsa-miR-1304. The risk score built by these multiple-type RNAs showed good performance to predict DFS, with an AUC of 0.954 and 0.882 in the training dataset and the testing dataset, respectively. The predictive accuracy of our signature was obviously higher than that of previously identified signatures for CC, such as 9-lncRNA (training GSE44001 dataset, AUC=0.793; testing TCGA dataset, AUC=0.742) (8) and 5-mRNA (TCGA dataset, AUC=0.792) (6). The predictive superiority of multiple-type RNA to single RNA type was also confirmed by our analysis (training: AUC=0.954 *vs* 0.659 for lncRNAs, 0.63 for miRNAs, 0.939 for mRNAs; testing: AUC=0.882 *vs* 0.602 for lncRNAs, 0.608 for miRNAs, 0.808 for mRNAs; training: C-index=0.855 *vs* 0.646 for lncRNAs, 0.628 for miRNAs, 0.806 for mRNAs; testing: C-index=0.711 *vs* 0.469 for lncRNAs, 0.606 for miRNAs, 0.703 for mRNAs). These findings were in agreement with the study of Zhang et al. (11) and Wang et al. (12). More importantly, we also found that the risk score had the ability for predicting the DFS within each FIGO stage and the AUC (training: 0.954 *vs* 0.501; testing: 0.882 *vs* 0.656) and C-index (training: 0.855 *vs* 0.539; testing: 0.711 *vs* 0.508) of the risk score were higher than those of the FIGO staging. Combination of the FIGO staging with the risk score did not obviously improve the predictive power, it even slightly decreased due to the fact that the FIGO staging was not an independent prognostic factor (data not shown). These conclusions were also observed in the study of Mao et al. (8). These findings indicated our risk score may be a more promising, effective, independent predictor for DFS in CC patients.

All our identified ceRNA axes were not reported previously, indicating they were novel insights to explain the mechanisms of recurrence and unfavorable prognosis in CC. However, the individual studies on these lncRNAs, miRNAs, and mRNAs may indirectly suggest their functions for CC. For example, Roychowdhury et al. (16) identified CD27-AS1 as a downregulated lncRNA in cervical carcinoma by microarray expression profile analysis. MiR-1275 was found to be upregulated in cancer cell lines and tissues of lung adenocarcinoma (17,18). High expression of miR-1275 was associated with shorter OS and RFS in lung cancer patients (17). Overexpression of miR-1275 promoted cancer cell migration, invasion, and proliferation (18), which may be related to its negative regulation on the downstream target genes (such as leucine zipper putative tumor suppressor 3) and then to enhance the stemness of cancer cells (17). Knockdown of ANKRD6 was revealed to increase melanoma cell proliferation and migration (19). Consistent with these studies, we also identified CD27-AS1 and ANKRD6 were downregulated, while miR-1275 was upregulated in recurrent CC tissues compared with non-recurrent controls. The HRs were less than 1 for CD27-AS1 and ANKRD6 and larger than 1 for miR-1275 in the DFS curve analysis (Supplementary Table S3), further indicating their tumor suppressor and oncogenic roles, respectively. Thus, theoretically, their related-ceRNA theory (that is, downregulated CD27-AS1 may be insufficient to sponge hsa-miR-1275 and then lead to the inhibitory effects of hsa-miR-1275 on ANKRD6) may be believable.

By analysis of the TCGA data, Liu et al. (20) showed that LINC00683 was significantly downregulated in prostate cancer samples, and high expression of LINC00683 was correlated with a more favorable OS compared with lower levels. MiR-146b expression was increased in CC tissues compared with paracancerous tissues (21). Down-regulation of miR-146b strongly suppressed proliferation, migration, and anchorage-independent growth of CC cells (21). The study of Sullivan et al. (22) reported that ACAP2 expression was downregulated in esophageal cancer, leukemias, and lymphoma. Knockdown of ACAP2 inhibited cancer cell apoptosis. These results implied that LINC00683 and ACAP2 exert tumor suppressor roles, while miR-146b was pro-oncogenic. This conclusion was also proven in our study, showing LINC00683 and ACAP2 were lower expressed, while miR-146 was higher expressed in recurrent CC tissues than those in the non-recurrent samples. Therefore, LINC00683-miR-146-ACAP2 ceRNA axis may also be a verifiable mechanism for CC.

Using the data from TCGA database, Zheng et al. (23) observed EMX2OS was significantly downregulated in the CC tissues compared with normal controls. K-M survival curve analysis showed CC patients with high expression of EMX2OS had better OS compared with the low-expression group. Univariate Cox analysis further validated that highly expressed EMX2OS was a protective factor for poor OS (HR=0.91). Upregulated expression of miR-1238 was indicated to confer chemoresistance for glioblastoma cells, while loss of miR-1238 sensitized resistant glioblastoma cells to temozolomide (24). The expression of miR-4648 was shown to be higher in the relapse cases of small cell carcinoma of the esophagus compared with non-relapse cases (25). The oncogenic activity of miR-29b-1-5p may result from the activation of epithelial-mesenchymal transition in cancer cells (26). Highly expressed miR-29b-1 was also proven to be closely correlated with shorter OS time in non-small cell lung cancer patients (27). High expression of FBLN5 was significantly correlated with better OS and DFS in hepatocellular carcinoma patients (28). A recent case report showed that GHR was very lowly expressed in patients with adamantinomatous craniopharyngioma and deficiency in growth hormone led to the development of recurrence at 18 months after surgery (29). Inhibition of MYLIP facilitated the growth and metastasis of CC cells (30). In accordance with these reports, we also identified that EMX2OS and FBLN5/GHR/MYLIP were downregulated, while hsa-miR-1238/4648/29b-1 was upregulated in recurrent CC tissues compared to those in the non-recurrent samples. Also, they were all DFS-related biomarkers. Accordingly, their-related ceRNA axes (EMX2OS-hsa-miR-1238-FBLN5, EMX2OS-hsa-miR-4648-GHR, EMX2OS-hsa-miR-29b-1-MYLIP) may be important targets for preventing the occurrence of recurrence in CC patients.

Several studies demonstrated that LINC00265 was significantly highly expressed in cancer patients and associated with poorer prognosis (31). Functional analysis revealed that depletion of LINC00265 impaired cell proliferation and invasion, promoted cell cycle distribution and apoptosis *in vitro*, and attenuated tumorigenesis *in vivo*(32). miR128-1 was suggested to function as a tumor suppressor because it was downregulated in glioblastoma multiforme and glioma stem-like cells. Forced expression of miR128-1 inhibited tumor cell proliferation, migration, and invasion *in vitro* and blocked the growth of transplant tumors *in vivo* (33). miR-604 also seemed to be a tumor suppressor miRNA due to low expression in cisplatin-resistant gastric cancer cells (34). miR-1304 was an independent risk factor for recurrence of esophageal carcinoma, showing that patients with high expression of miR-1304 had a relatively lower survival rate (35). Patients with a loss of ATRX expression were found to have longer survival times (36). ARMC7 was found to be amplified across several cancer tissues and cell lines (37). OXCT1, which encodes a 3-oxoacid CoA transferase 1 enzyme necessary for ketone catabolism and then involving in the tricarboxylic acid cycle, was also expressed at higher levels in metastatic colorectal cancer cell lines (38). Similarly, RAB39A was highly expressed in sarcomas and in malignancies of lymphoid, adrenal, and testicular tissues. RAB39A may facilitate tumorigenesis by interaction with its downstream RXRB (39). In agreement with these findings, we also identified LINC00265 and ATRX/ARMC7/OXCT1 were upregulated in recurrent CC tissues compared with those of the non-recurrent samples and were risk factors for poor DFS (HR>1), while hsa-miR128-1/604/1304 were downregulated and protective factors for DFS (HR<1). Hence, we predicted that LINC00265 may be responsible for the recurrence and the malignant phenotype of CC patients by functioning as a ceRNA via sponging hsa-miR128-1/604/1304 to elevate the expressions of ATRX/ARMC7-OXCT1/RAB39A.

There were some limitations in this study. First, the prognostic signature was identified and validated based on bioinformatics analyses of retrospective public data. The prognostic value of our molecular model needs to be further validated using larger clinical CC samples prospectively collected from multiple hospitals. Second, the identified ceRNA interactive axes of prognostic genes should be demonstrated by *in vitro* or *in vivo* experiments. Third, the roles of other genes that were not significantly associated with RFS but were differentially expressed in our study and previous reports (such as hsa-miR-196a) (40) require further investigation. Fourth, in addition to ceRNA, lncRNAs also could function by directly binding with mRNAs (14). mRNAs did not independently play important key roles in cancer, but by interaction with each other (10). In subsequent studies, the lncRNA-mRNA co-expression network or protein-protein interaction network should be established to find other underlying prognostic signatures for CC.

### Conclusion

In this study, we developed a novel risk score model based on two-lncRNA-three-miRNA-seven-mRNA signature for CC patients. This signature not only discriminated patients at a high risk of recurrence from patients at a low risk of recurrence, but also accurately predicted the outcomes of DFS for CC. Its predictive power for DFS was higher than that of the FIGO staging and single RNA-type model. This model was derived from genes in the ceRNA network and, thus, this study sheds new light on the molecular mechanisms of tumorigenesis and provided promising therapeutic targets for CC patients.

## Supplementary Material

Click to view [zip].
